# Comparison of different site preparation techniques: quality of planting spots, seedling growth and pine weevil damage

**DOI:** 10.1007/s11056-018-9634-8

**Published:** 2018-03-07

**Authors:** Kristina Wallertz, Niklas Björklund, Karin Hjelm, Magnus Petersson, Lars-Göran Sundblad

**Affiliations:** 1 Asa Forest Research Station, Swedish University of Agricultural Sciences, Lammhult, Sweden; 2 Department of Ecology, Swedish University of Agricultural Sciences, Box 7044, 750 07 Uppsala, Sweden; 3 The Forestry Research Institute of Sweden, Skogforsk, Ekebo 2250, 26890 Svalöv, Sweden; 4 Södra Forest Owner Association, Skogsudden, 351 89 Växjö, Sweden

**Keywords:** Hylobius abietis, Pine weevil, Planting, Site preparation techniques

## Abstract

In northern Europe, there are high risks of severe pine weevil (*Hylobius abietis*) damage to newly planted conifer seedlings. Site preparation is one of the most important measures for reducing these risks and as several studies have shown the damage is highly dependent on the amount of pure mineral soil around the seedlings. We investigated effects of three site preparation techniques: (1) disc trenching with a conventional Bracke T26, (2) MidiFlex unit and (3) soil inversion with a Karl Oskar unit on characteristics of the planting spots, growth and pine weevil damage and survival rates of untreated and insecticide treated planted Norway spruce (*Picea abies*) seedlings. All three site preparation techniques reduced pine weevil damage in comparison with no site preparation, and the proportion of spots with pure mineral soil they created was inversely related to the rate of mortality caused by pine weevil. The results indicate that the quality of the planting spots depends on the technique used. In areas where pine weevil is the major threat to seedling survival, the amount of mineral soil in the planting spots is the most important factor in order to protect the seedling from damage. Without site preparation most planting spots consisted of undisturbed humus. Generally, the Karl-Oskar created the most spots with pure mineral soil, but on very stony soils the Bracke T26 created more mineral soil spots than other methods. Site preparation is a valuable tool in order to improve survival in regeneration areas and it is of great importance to make the right choice of technique depending on the particular circumstances on the actual site.

## Introduction

The management of vegetation competition in forests is an integral part of silvicultural practices in many parts of the world (Wagner et al. 2006; Richardson et al. 2006; Newton 2006). Mechanical site preparation is therefore used in many countries in order to reduce competition from other vegetation (Löf et al. 2012; Nilsson and Örlander 1999a, b; Thiffault and Jobidon 2006), but also to improve growing other conditions for newly planted seedlings (Sutton and Bedford 2001; Petersson et al. 2005; Luoranen and Viiri 2012). The improvement is due to interactive effects of many factors that facilitate seedlings’ establishment. These include alterations of the microenvironment that increase nutrient availability (Munson et al. 1993; Schmidt et al. 1996. Grossnickle 2000) and beneficial changes in both soil moisture and temperature (Folk and Grossnickle 2000).

Another, often crucial factor is that mechanical site preparation can affect the frequency and severity of attacks by pine weevils (*Hylobius abietis*), the most serious pests of planted conifer seedlings on forest land in northern Europe (Leather et al. 1999; Örlander and Nilsson 1999; Långström and Day 2004), The seedlings’ survival is highly dependent on the degree of suppression of damage caused by the weevils. To reduce damage, large proportions of conifer seedlings are treated with insecticides, or various coatings or feeding barriers. However, at most sites there is high feeding pressure from pine weevils, and at least two measures must be used to protect seedlings sufficiently (Petersson and Örlander 2003). If no measures are applied to protect newly planted seedlings, up to 80% of them may die during the first 3 years (von Sydow 1997). Several studies have shown that site preparation methods can reduce the damage caused by pine weevils (Lekander and Söderström 1969; von Sydow 1997; Örlander and Nilsson 1999; Sutton; 1993), especially if they result in planted seedlings being surrounded by pure mineral soil (Lindström et al. 1986; Björklund et al. 2003; Petersson and Örlander 2003; Petersson et al. 2005). Pine weevils seem to avoid areas of mineral soil by increasing their speed and moving straighter. This reduces the time they remain on the patch resulting in less feeding on those seedlings compared to seedlings planted in humus (Kindvall et al. 2000).

In practice, planting spots’ quality varies considerably depending on site conditions, site preparation method and equipment used but the result could also be affected by the skills and motivation of the operators.

Disc-trenching is a common method world-wide in planted forest (Sutton 1993), and is currently the most commonly used in Sweden (Bergquist et al. 2011). Other methods used in several countries are for example blading, scalping, mixing, mounding and inverting (McMinn 1983; Hallsby and Örlander 2004). Inverting (lifting the soil profile at each planting spot, turning it upside down, and replacing it at the same spot) has in several studies proved to improve seedling survival and increase growth rates (Örlander et al. 1998; Granhus et al. 2003; Johansson et al. 2013). Currently, an excavator is used for most inverting, but new technique is developing and might increase the use of the method in the future. New types of disc trenchers have also been introduced. For example, the MidiFlex (Midiflex AB) creates similar trenches and berms to a conventional disc-trencher, but due to differences in its construction the rear boogie runs over and compresses the berms. Thus, the berms are less loose and fluffy, and more suitable for immediate planting than berms created by conventional aggregates, which must settle over a winter season to avoid the presence of air pockets when seedlings are planted.

The aim of this study was to investigate effects of three site preparation techniques (disc trenching with a conventional Bracke T26 or MidiFlex unit and soil inversion with a Karl Oskar unit) on characteristics of the planting spots, and growth, pine weevil damage and survival rates of untreated and insecticide treated Norway spruce (*Picea abies*) seedlings.

A control treatment (no site preparation) was also included. Various studies have compared seedling growth and survival rates following site preparation by different techniques, but few have also compared the nature of the planting spots.

For example, we wanted to test if a site preparation technique that can adjust the planting spots according to differences in soil moisture conditions and stoniness can generate a higher proportion of high quality spots.

In this study area the quality of the planting spot was correlated to the achieved amount of mineral soil in order to reduce the risk of damage by pine weevil.

Classification of planting spots, the effect of site preparation technique on sites with various stoniness and soil type class, as well as the effects on seedling height growth and survival rates could be of high interest for any other countries that uses site preparation as a regeneration tool.

## Materials and methods

### Experimental sites

**Fig. 1:**
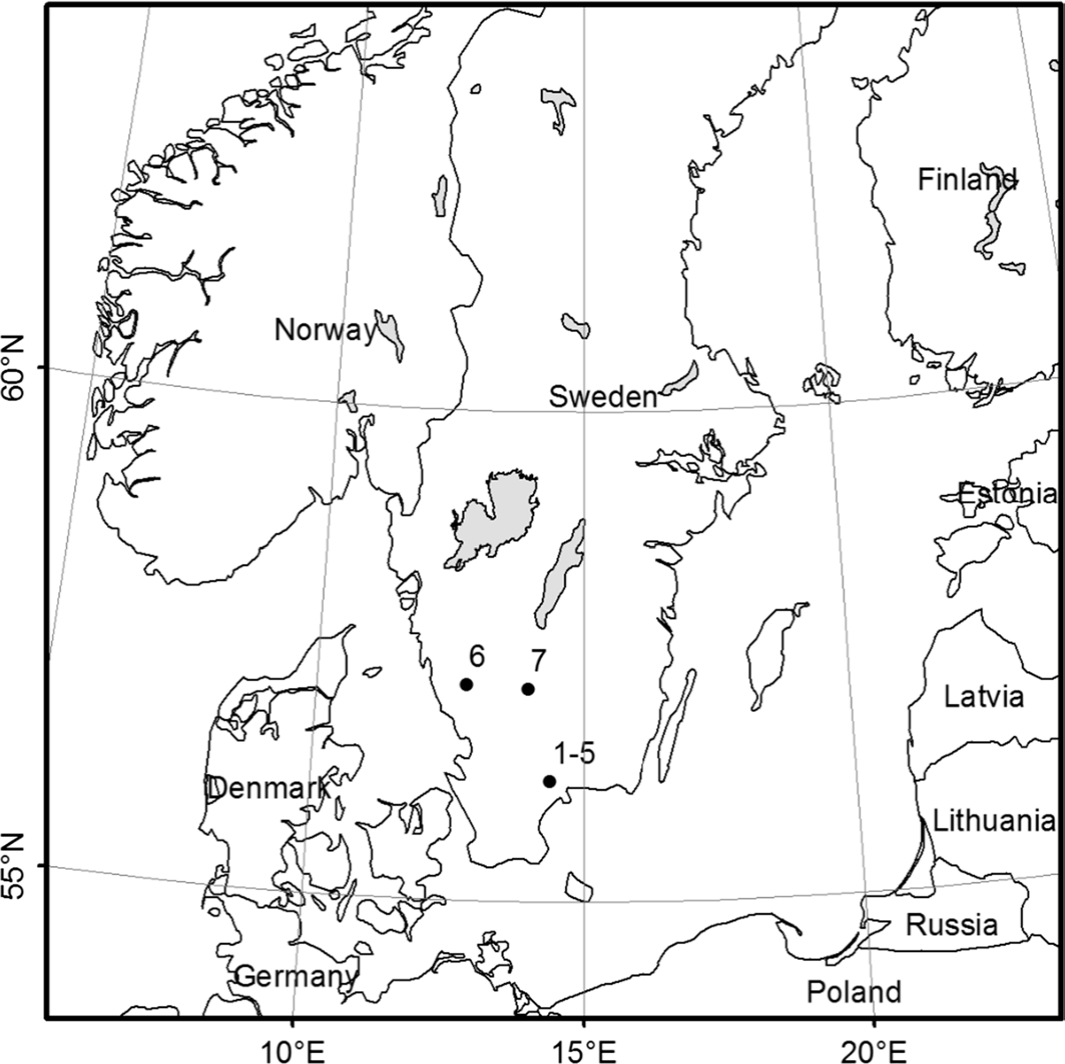
Locations of the seven experimental forest sites (1–7)

Seven experimental forest sites were used in this study. They were clear-cut during the winter of 2010–2011 or 2011–2012 and planted during the following spring (Table 1, Fig. 1) and the area varied between 0.8 and 7.0 ha. Sites 1–5 were located in the same area, all within a radius of 35 km, in the province of Scania (56°13′N, 14°23′E), site 6 was located in the province of Västergötland, just 40 km from the west coast (57°9′N, 13°58′E) and site 7 inland, in the province of Småland (57°11′N, 12°48′E) (Fig. 1). The sites were representative of relatively fertile forest sites, supporting tree growth rates of around 10 m^3^ ha^−1^ year^−1^, with original stands dominated by Norway spruce (*Picea abies* L. Karst.) Sandy silt was the most common soil texture, and only two of the sites consisted of sandy till. The mean annual precipitation (data from 1960 to 1990) was 629 mm for the sites in Scania (1–5) and 1032 and 812 mm respectively for Västergötland (site 6) and Småland (site 7). The mean temperature (data from 1962 to 1990) was 6.6, 5.8 and 6.1 °C respectively. (Data were received from SMHI, the Swedish Meteorological and Hydrological Institute. We extracted the mean annual precipitation and mean annual temperature for each NFI plot using ArcGis version 10.3).

**Table 1:** Frequency distributions (%) of planting spots classified as no scarification, track/patch, mound/berm and inverted, created by each unit (T26 = Bracke T26, MF = MidiFlex and KO = Karl Oskar) or with no site preparation (No SP), at micro-sites assigned to each of the three soil moisture classes (dry, mesic and moist)

Site prep	Soil moisture	Percentage of planting spot classified (%) in brackets (n)			
		No Scarification	Track/patch	Mound/berm	Inverted
No SP	Dry	100 (n = 59)	0 (n = 0)	0 (n = 0)	0 (n = 0)
	Mesic	99 (n = 653)	1 (n = 3)	0 (n = 0)	0 (n = 0)
	Moist	100 (n = 25)	0 (n = 0)	0 (n = 0)	0 (n = 0)
T26	Dry	0 (n = 0)	100 (n = 42)	0 (n = 0)	0 (n = 0)
	Mesic	1 (n = 7)	91 (n = 615)	8 (n = 55)	0 (n = 0)
	Moist	0 (n = 0)	100 (n = 21)	0 (n = 0)	0 (n = 0)
MF	Dry	6 (n = 3)	87 (n = 47)	7 (n = 4)	0 (n = 0)
	Mesic	4(n = 29)	29 (n = 192)	66 (n = 434)	0 (n = 0)
	Moist	7 (n = 2)	66 (n = 20)	27 (n = 8)	0 (n = 0)
KO	Dry	0 (n = 0)	24 (n = 13)	38 (n = 20)	38 (n = 20)
	Mesic	0 (n = 0)	10 (n = 60)	22 (n = 142)	68 (n = 434)
	Moist	2 (n = 1)	12 (n = 6)	80 (n = 41)	6 (n = 3)

In the parenthesis after the percentage the total number of planting spots in each class is given within each category (= n)

### Seedling material

The Norway spruce seedlings were obtained from a commercial nursery, Södra Odlarna, Falkenberg (Sweden) and originated from Bredinge seed orchard located in southern Sweden (56°48′N, 16°43′E). They were grown for 2 years in 90 cm^3^ HIKO^®^ multi-pot containers, which had an internal coating of copper sulphate to reduce root deformation. Half of the seedlings were left untreated whereas the other half were treated at the nursery with Merit Forest WG insecticide (Bayer, active ingredient imidacloprid, 1.4%; prepared by mixing 14 g of the granulated material per litre of water).

### Site preparation techniques

Planting spots (except the controls) were prepared at each study site using a Bracke T26 disc trencher (Bracke Forest AB, Bräcke, Sweden), a Midiflex disc trencher (Midiflex AB, Alfta produkter AB, Alfta, Sweden), both mounted on forwarders and a Karl Oskar (BSM Verkstads AB, Alvesta, Sweden) unit mounted on an excavator. For practical reasons different persons were hired to perform the site preparations at the different occasions, i.e. in total three operators for Bracke T26, one operator for Midiflex and two operators for Karl Oskar.

The Bracke T26 is a conventional two-row, rear end-mounted disc trencher, designed to create tracks or furrows and berms of inverted soil (from the tracks) covering the soil beside them, so the surface of the berms consists of mineral soil. The Midiflex is also a two-row disc trencher but with the unit and discs mounted in the middle of the forwarder in front of the rear boogie. It creates rows of track and berms, like a conventional disc trencher, but the rear bogie runs over and thus compresses the berms. The Karl Oskar is an excavator-based preparation unit that is specially designed for creating various kinds of planting spots. The operator can use it for either inverting, mounding or patch scarification. Thus, the method applied to create each planting spot can be adapted in accordance with the characteristics of the spot and seedling to be planted.

### Experimental design

**Fig. 2:**
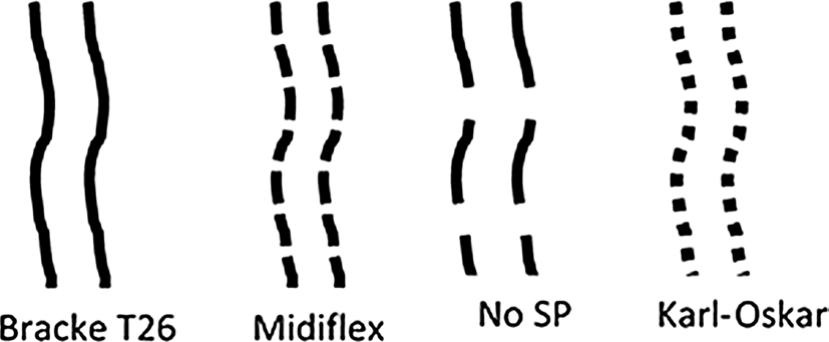
Schematic illustration of the experimental design. Each machine created two tracks of planting spots, with the order randomly distributed between the four treatments: site preparation by the three machines and no site preparation (NO SP)

At each site, rows of planting spots were created by each of the three machines, in blocks of six prepared tracks, two per machine, and two tracks with no site preparation (Fig. 2).

The order in which the machines made the tracks was randomly selected. Each block covered approximately 20 × 20 m, and their positions were intended to ensure that site conditions were as homogeneous as possible within them and the soil moisture in each block was classified as one of three classes: dry, mesic or moist (Hägglund and Lundmark 1987). The number of blocks created at the sites varied between 2 and 14, depending on sizes of the clear-cuts and suitable areas for planting.

In each block, two rows of 20 seedlings (10 that had been treated with the insecticide and 10 that had not) were planted in spots created by each treatment. Thus, 80 seedlings were planted in each block (at ca. 2 m spacing), and there were 37 blocks in total, so 2960 seedlings were used in the experiment.

The following instructions were given to the operator of each machine and the staff planting the seedlings.

1. *No site preparation* avoid planting in low spots.
2. *Bracke T26* On dry or mesic soil, the operator should try to create shallow tracks with mineral soil surfaces and the seedlings should be planted in the middle of them. If a spot in the middle of a track is too low so there is a risk of standing water and drowning of the seedling as a consequence, the seedling should be planted at a higher level. On moist soil, the driver should still aim to create tracks of mineral soil, but the seedlings should be planted in the area between the track and the berm (the so-called “hinge”) rather than in the middle of the track.
3. *Midiflex* The operator should try to get plenty of mineral soil on the compressed berms and the seedlings should be planted on them. If there is insufficient mineral soil on the berm, they should be planted in the track, but avoiding low spots. If no planting spots containing mineral soil are created, the seedlings should be planted in the berms.
4. *Karl Oskar* The operator was instructed to select an appropriate type of preparation depending on the stoniness and other site conditions: mounds should be made in moist spots, inverted spots in all other areas where possible, and patch spots otherwise.

Stoniness was assessed by pushing a metal pin into the ground to a depth of 20 cm (Berg and Wickström 1982) at 20 randomly chosen points in each block and recording the number of times it hit a stone. Stoniness was then simply expressed as the number of ‘hits’ divided by 20, the number of points. The stoniness of the blocks varied between 20 and 85% and was on average 50%.

### Seedling level measurements

The site preparation results within a 1 m radius around each seedling, i.e. the planting spot were examined directly after planting, and recorded as predominantly: no scarification (undisturbed humus); track or patch (exposed mineral soil); mound or berm (with mineral soil on top); or inverted (soil profile lifted, turned upside down and replaced). The soil moisture within this area was also recorded, as dry, mesic or moist.

Within a radius of 10 cm around each seedling the dominating soil type, i.e. soil type class was determined in four classes: undisturbed humus, disturbed humus, mixture of humus and mineral soil, and pure mineral soil. The height of each planting spot was measured to the closest centimetre, relative to the undisturbed ground bordering the site preparation spot, and could be negative if the spot was lower than the undisturbed ground. The shortest distance (cm) from the seedling to disturbed humus (referred to as the minimum distance between mineral soil and the seedling) was measured, to the closest centimetre, at the same time.

Directly after planting, the height of the aboveground part of each seedling was measured, in late autumn (October) during each of the first 3 years, we registered its height, length of its leading shoot and damage by pine weevil the length measures were done to the closest centimetre and the debarked area of the stem to the closest 0.1 cm^2^. The severity of damage was recorded subjectively using a six-level scale: 0 = undamaged, 1 = slightly damaged, 2 = uncertain or insignificant damage, 3 = severely damaged, 4 = life-threatening damage, and 5 = dead. Damage due to other causes (fungi, frost, drought, waterlogging, competing vegetation, and browsing) was also determined.

### Statistical analyses

The outcome of the site preparation treatments was described in terms of frequencies and percentages of planting spots in recorded categories. For this, the FREQ procedure in SAS 9.4 was used (SAS Institute, Cary, NC, USA). Chi square tests were used to assess statistical differences between site preparation methods. Effects of stoniness of the ground on site preparation results, in terms of soil type class and distance from undisturbed humus in planting spots were also analyzed. A first-order regression model, with soil type class or distance from undisturbed humus as the response variable, and stoniness as the explanatory variable, was applied using PROC GLM in SAS 9.4. In the model, site preparation treatment was included as a qualitative predictor variable and the effect of interaction between stoniness and site preparation was tested. For example, the model used to investigate the effects of the four site preparation treatments was:

1 $${\text{Y}}_{\text{i}} =\upbeta_{0} + \,\upbeta_{1} {\text{X}}_{{{\text{i}}1}} + \,\upbeta_{2} {\text{X}}_{{{\text{i}}2}} + \,\upbeta_{3} {\text{X}}_{{{\text{i}}3}} + \,\upbeta_{4} {\text{X}}_{{{\text{i}}4}} + \,\upbeta_{5} {\text{X}}_{{{\text{i}}1}} {\text{X}}i_{2} + \,\upbeta_{6} {\text{X}}_{{{\text{i}}1}} {\text{X}}i_{3} + \,\upbeta_{7} {\text{X}}_{{{\text{i}}1}} {\text{X}}i_{4} + \,\upvarepsilon_{\text{i}}$$

where Y = soil type or distance from undisturbed humus, β_0_ is the intercept and β_1_–β_7_ the regression coefficients, X_1_ is the stoniness and the last X variables represent the indicator variables for the four site preparation treatments (see Neter et al. 1996).

When analysing seedling height (cm), and length of leader (cm) data recorded for all seedlings classified as living seedlings were used. The mean area debarked (cm^2^) in each year was analyzed using data for all living seedlings, and seedlings that were attacked and killed during that year.

The accumulated debarked area during the experimental period was based on all planted seedlings. Both height and debarked area adequately followed a normal distribution with equal variances, and were analysed with a mixed model implemented in PROC MIXED in SAS 9.4:

2 $${\text{Y}}_{\text{ijklm}} = \,\upmu \, + {\text{ a}}_{\text{ir}} + {\text{ b}}_{\text{jk}} + \,\upgamma_{\text{l}} + \,\updelta_{\text{m}} + \,\upgamma_{\text{l}}\updelta_{\text{m}} + \,\upvarepsilon_{\text{ijklm}}$$

where µ is the general mean, a_i_ is the random effect of site (i = 1–7), b_jk_ is the random effect of block (j = 1–21) within planting year (k = 1–2), γ_l_ is the fixed effect of site preparation treatment (l = 1–4) and δ_m_ is the fixed effect of insecticide treatment (m = 1–2). The interaction between site preparation and insecticide treatment was included in the model as a fixed effect. To test effects of soil type on seedling height, length of leader and debarked area 3 years after planting, the same model was used, but instead of site preparation the fixed effect of the four soil type classes was tested. A similar model was used to test for differences in height of planting spot and distance to disturbed humus between site preparation methods and planting spot class. Satterthwaite approximation was used to determine appropriate degrees of freedom in all analyses. When significant differences between treatment means were detected, they were separated by least square means, with Tukey–Kramer adjustment of p-values. An α-level of 0.05 was used in all analyses.

When analysing effects of the explanatory variables on the proportion (η) of attacked or killed seedlings after the first three growing seasons, the input data adequately followed a binomial distribution to use generalized linear mixed models implemented in PROC GLIMMIX in SAS 9.4. Total seedling mortality was defined as the proportion of planted seedlings that had been killed at the end of the third year. The proportion of seedlings that had been attacked by pine weevil was analysed both per year and as an accumulated value over the experimental period. For these analyses, a logit link function was used:

3 $${\text{logit }}\left( {\upeta_{\text{ijkml}} } \right) \, = { \log }\left( {\upeta_{\text{ijklm}} /\left( {1 - \,\upeta_{\text{ijklm}} } \right)} \right) \, =\upmu \, + {\text{ a}}_{\text{i}} + {\text{ b}}_{\text{jk}} + \,\upgamma_{\text{l}} + \,\updelta_{\text{m}} + \,\upgamma_{\text{l}}\updelta_{\text{m}} + \,\upvarepsilon_{\text{ijklm}}$$

where the explanatory variables were the same as those in Eq. 1.

## Results

### Site preparation

Without site preparation, almost all planting spots were classified as “no scarification (undisturbed humus)”. After preparation using the Bracke T26, most planting spots were classified as tracks or patches, regardless of soil moisture class. Of spots created by the MidiFlex, most were mounds/berms and about a third were tracks/patches. As expected, many spots created by the Karl Oskar were classified as inverted, but the results also reflected the instructions and the unit’s capacity to create diverse kinds of planting spots in accordance with site conditions. In dry blocks, the planting spots were almost evenly distributed between tracks/patches, mounds/berms and inverted. In mesic blocks most spots were inverted, while in moist blocks they were mostly mounds/berms (Table 1).

Planting spots were also classified according to their soil type and the distribution of soil type classes differed significantly between the site preparation methods (*p* = 0.0001 for all soil type classes). Without site preparation, almost all planting spots (94%) consisted of undisturbed humus (Table 2). Most of the planting spots created by the Bracke T26 consisted of pure mineral soil (47%) a mixture of humus and mineral soil (35%), or disturbed humus (18%). The MidiFlex created the highest proportion of planting spots with disturbed humus (36%), and correspondingly lower proportions of spots with pure mineral soil or a mixture of humus and mineral soil than the Bracke T26 (22 and 38%, respectively). The Karl Oskar created more spots with pure mineral soil, 67% on average, and this was significantly higher compared with the other site preparation methods. Of the inverted planting spots it created, 79% consisted of pure mineral soil.

**Table 2:** Frequency distributions (%) of soil type classes among planting spots of each class and overall (bold style) created by each unit or with no site preparation (No SP)

Site prep	Planting spot	Soil type class among planting spots %			
		Undisturbed	Disturbed	Mixture	Mineral soil
No SP	No scarification	96 (n = 704)	3 (n = 27)	1 (n = 1)	0 (n = 0)
	Tracks	0 (n = 0)	100 (n = 1)	0 (n = 0)	0 (n = 0)
	Overall	96 (n = 704)	4 (n = 28)	0 (n = 1)	0 (n = 0)
T26	No scarification	86 (n = 6)	14 (n = 1)	0 (n = 0)	0 (n = 0)
	Tracks	0 (n = 0)	18 (n = 123)	35 (n = 234)	47 (n = 321)
	Berm	0 (n = 0)	11 (n = 6)	40 (n = 22)	49 (n = 27)
	Overall	1 (n = 6)	18 (n = 130)	35 (n = 256)	47 (n = 348)
MF	No scarification	82 (n = 27)	15 (n = 5)	3 (n = 1)	0 (n = 0)
	Tracks	0 (n = 0)	34 (n = 89)	45 (n = 115)	21 (n = 54)
	Berm	1 (n = 2)	38 (n = 171)	36 (n = 163)	25 (n = 110)
	Overall	4 (n = 29)	36 (n = 265)	38 (n = 279)	22 (n = 164)
KO	No scarification	100 (n = 1)	0 (n = 0)	0 (n = 0)	0 (n = 0)
	Patch	0 (n = 0)	23 (n = 18)	44 (n = 35)	33 (n = 26)
	Mound	0 (n = 0)	8 (n = 16)	41 (n = 82)	51 (n = 104)
	Invert	0 (n = 0)	2 (n = 9)	19 (n = 85)	79 (n = 363)
	Overall	0(n = 1)	6 (n = 6)	27 (n = 202)	67 (n = 493)

In the parenthesis after the percentage the total number of planting spots in each class is given within each category (= n)

*T26* Bracke T26, *MF* MidiFlex, *KO* Karl Oskar, *Undisturbed* undisturbed humus, *Disturbed* disturbed humus, *Mixture* mixture of humus and mineral soil, *Mineral soil* pure mineral soil, *Tracks* tracks and patches, *Berm* berms and mounds, *Invert* inverted spot

**Fig. 3:**
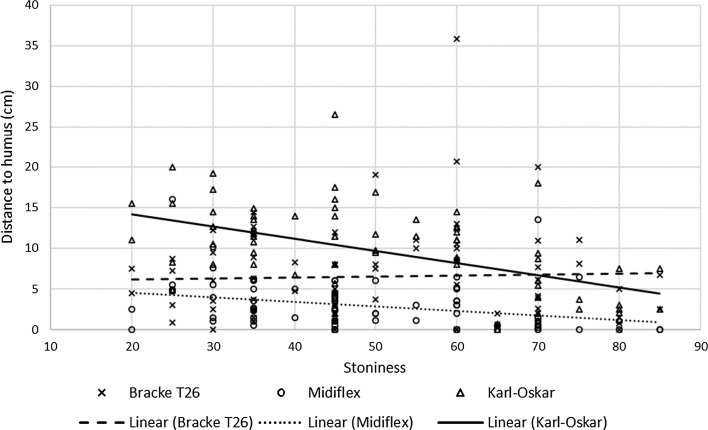
Effects of stoniness on site preparation results in terms of distance to disturbed humus in planting spots. Crosses represent planting spots after Bracke T26, open circels MidiFlex and open triangles Karl-Oskar. The dashed line (Bracke T26), dotted line (MidiFlex) and solid line (Karl Oskar) indicate linear regression lines of results obtained with the respective aggregates

The height of the planting spot (topography) and shortest distance from the seedling to disturbed humus were also affected by the site preparation treatment and planting spot class (Table 3). Planting spots in tracks and patches were at a similar level to the surrounding ground, while those in mounds/berms were up to more than 10 cm higher on average than the surrounding ground. The distance from disturbed humus was longest in inverted spots created by the Karl Oskar, followed by mounds/berms created by the Karl Oskar or Bracke T26, and generally shortest in spots created by the MidiFlex (Fig. 3).

**Table 3:** Height relative to surrounding ground and distance to disturbed humus of planting spots of each class created by each unit (mean values)

Site preparation	Planting spot class	Height (cm)	Distance to disturbed humus (cm)
T26	No scarification (n = 7)	4.57^b^	0^a^
	Track/patch (n = 678)	0.023^a^	6.50^b^
	Mound/berm (n = 55)	10.16^c^	7.45^b^
MF	No scarification (n = 34)	4.5^b^	0^a^
	Track/Patch (n = 259)	1.69^a^	2.60^a^
	Mound/Berm (n = 446)	6.47^b^	3.15^ab^
KO	Track/Patch (n = 79)	− 0.22^a^	2.75^ab^
	Mound/Berm (n = 203)	5.85^b^	7.66^b^
	Inverted (n = 457)	7.61^b^	11.78^c^

*T26* Bracke T26, *MF* MidiFlex, *KO* Karl Oskar

Different letters indicate significant differences

**Fig. 4:**
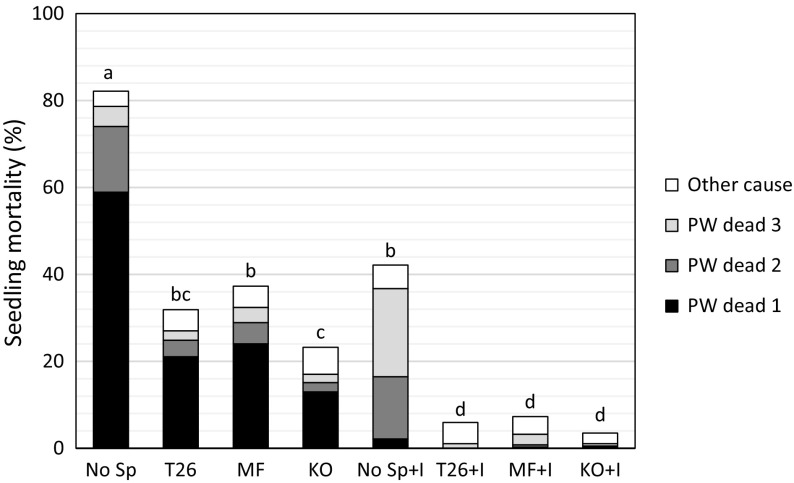
Seedling mortality after three growing seasons under indicated combinations of treatments: mortality caused by pine weevils in years 1, 2 and 3 after planting, and accumulated mortality caused by other factors.* No Sp* no site preparation;* T26, MF and KO* site preparation with the Bracke T26, MidiFlex and Karl Oskar units, respectively;* + I * insecticide treatment. Different letters above columns indicate significant differences in total mortality which are obtained by differences of least square means adjusted to Tukey–Kramer

The effect of stoniness on distance to disturbed humus was also tested (Fig. 4). With a coefficient of determination (R^2^) of 0.48, the model did not fit the data as well as the model describing the effect of stoniness on soil type class (R^2^ = 0.91). However, it revealed the same patterns, and both models detected highly significant effects on site preparation (*p* < 0.001). The Karl Oskar created spots with the longest distances between planted seedlings and disturbed humus, but as stoniness increased, these distances decreased. In areas with a stoniness index ≥ 70, the Bracke T26 created planting spots with longer distances to disturbed humus than the other units.

### Effects of site preparation on seedling mortality and attack rates

**Fig. 5:**
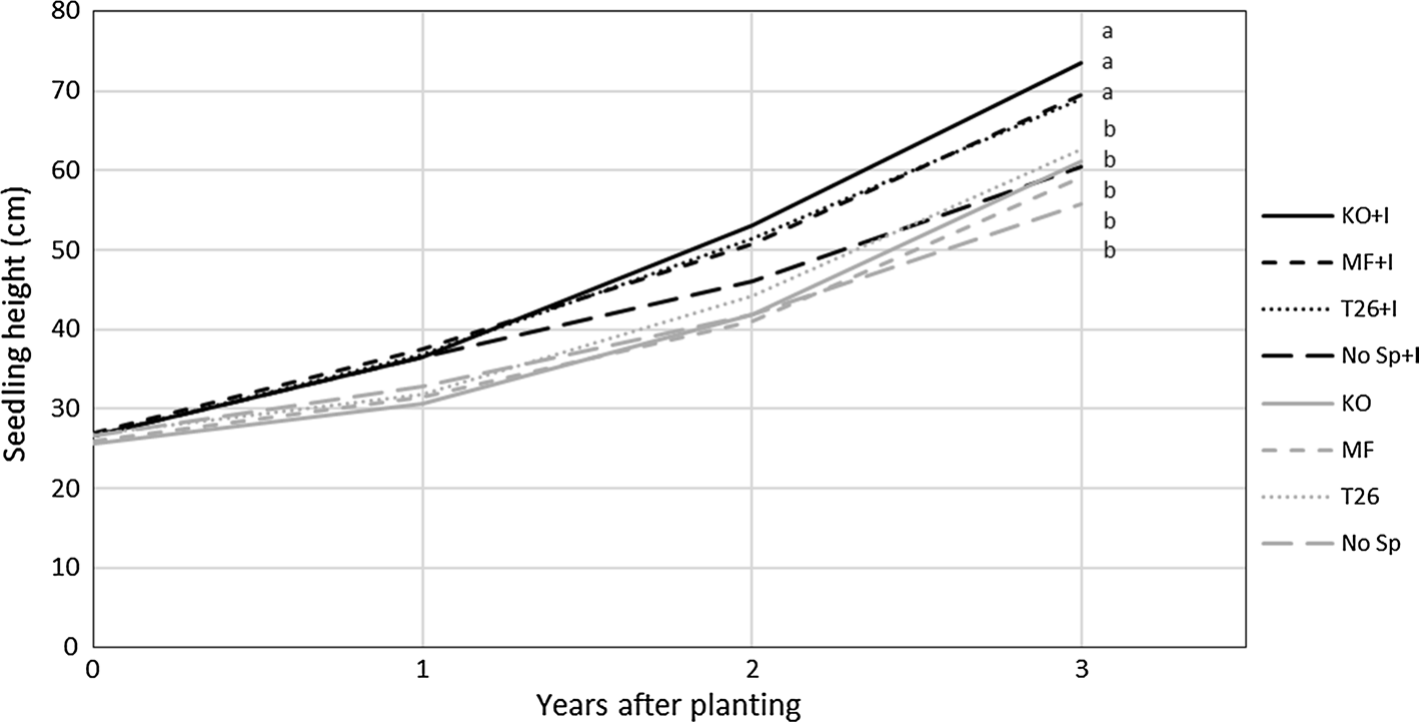
Average seedling height (cm) under indicated combinations of treatments during the 3 years following planting.* No Sp*  no site preparation;* T26, MF and KO* site preparation with the Bracke T26, MidiFlex and Karl Oskar units, respectively;* + I * insecticide treatment. Different letters indicate significant differences which are obtained by differences of least square means adjusted to Tukey–Kramer

Most of the seedling mortality caused by pine weevils occurred during the first growing season, but a substantial amount of seedlings were also killed during the second and third year in some of the treatments (Fig. 4). Seedlings planted in plots treated with any of the site preparation machines had a substantially lower mortality rate than seedlings planted without site preparation (*p* < 0.0001). After 3 years, the overall rates of mortality caused by pine weevils, including both untreated and treated seedlings, were 9, 14, and 18% following site preparation with the Karl Oskar, Bracke T26 and MidiFlex units, compared with 58% following no site preparation. On average, after 3 years 11 and 39% of the seedlings treated and not treated with insecticides had been killed, respectively, and the difference was statistically significant (*p* < 0.0001). A significant interaction between the site preparation and insecticide treatments was detected (*p* = 0.0039), showing that the Karl Oskar reduced the mortality of seedlings that had not been treated with insecticide more than the other units (Fig. 5). Mortality of seedlings caused by factors other than pine weevils was low; < 5% on average (Fig. 4). The total mortality, including both mortality caused by pine weevils and other factors was significantly different between site preparation treatments and insecticide treatment (*p* < 0.0001 for both) after 3 years.

Most of the seedlings (80.3%) were attacked, but not necessarily killed, by pine weevils during the experimental period. During the first growing season, both the insecticide treatment and site preparation strongly reduced attack rates (Table 4). The Karl Oskar and Bracke T26 units had significantly stronger effects in this respect than the MidiFlex. The area debarked during the year followed the same pattern, except that the Karl Oskar and Bracke T26 had non-significantly stronger effects than the MidiFlex (Table 4).

**Table 4:** Frequency of attacked seedlings (Attack,  %) and mean debarked area (Debark, cm^2^) under indicated combinations of treatments in each of the 3 years following planting and accumulated values over the experimental period (Total)

Treatment combination	Year 1		Year 2		Year 3		Total	
	Attack	Debark	Attack	Debark	Attack	Debark	Attack	Debark
No Sp	87^a^	7.3^a^	70^ab^	4.3^b^	27^a^	2.0^a^	97^a^	9.6^a^
T26	36^c^	2.1^b^	61^cd^	1.9^cd^	13^b^	0.4^c^	76^cd^	3.5^bc^
MF	49^b^	2.5^b^	63^bcd^	2.4^c^	19^b^	0.7^c^	84^b^	4.6^b^
KO	30^c^	1.6^b^	54^d^	1.3^d^	16^b^	0.5^c^	77^cd^	2.9^cd^
No Sp + I	56^b^	1.6^b^	83^a^	7.1^a^	30^a^	1.6^b^	92^a^	9.5^a^
T26 + I	10^e^	0.1^c^	67^bc^	2.4^c^	13^b^	0.4^c^	73^cd^	2.9^cd^
MF + I	20^d^	0.3^c^	73^b^	2.9^c^	16^b^	0.4^c^	80^bc^	3.5^bc^
KO + I	8^e^	0.1^c^	63^bcd^	2.2^cd^	15^b^	0.4^c^	69^d^	2.5^d^
Site preparation	0 0001	0.0001	0.0001	0.0001	0.0001	0.0001	0.0001	0.0001
Insecticide	0.0001	0.0001	0.0001	0.0001	0.1336	0.0883	0.0003	0.0457
Site prep × I	0.7645	0.0001	0.8841	0.0480	0.6995	0.3701	0.2004	0.6473

*No Sp*  no site preparation; *T26, MF and KO* site preparation with the Bracke T26, MidiFlex and Karl Oskar units, respectively;* + I * insecticide treatment

Different letters indicate significant differences

The three last lines shows the effects (*p* values) of site preparation, insecticide treatment (Insecticide) and their interaction on pine weevil induced attack rates (according to PROC GLIMMIX modelling) and debarked areas of seedlings (according to PROC MIXED modelling) during and after the first three growing seasons of the experiment

During the second growing season the insecticide treatment had the opposite effect, as more seedlings treated with insecticide at the time of planting were attacked and debarked than the others. However, this increase in attack frequency did not lead to an increase in seedling mortality in the second year (Fig. 4). During the third growing season, no significant effects of insecticide treatment were detected, but there was still an effect of site preparation, as attack frequencies and areas debarked were highest in plots that had received no site preparation (Tables 5, 6). At the end of the experiment, there were still differences in accumulated numbers of attacked seedlings and total debarked area between plots subjected to different site preparation and insecticide/no insecticide treatments, although the overall attack levels were high. After 3 years, regardless of whether the seedlings had been treated with insecticide or not, all site preparation treatments had reduced both the proportion of seedlings attacked by pine weevils and the debarked area in comparison with no site preparation. However, the Karl Oskar had resulted in significantly larger reductions than the MidiFlex (Table 5).

**Table 5:** Effects of site preparation, the insecticide treatment and their interaction (*p* values) on seedling height and length of leader after the first three growing seasons (1, 2 and 3), according to PROC MIXED modelling

Parameter	Effect		
	Site prep	Insecticide	Site prep × insecticide
Height at planting	0.4378	0.1297	0.3554
Height year 1	0.5797	< 0.0001	0.4852
Length of leader 1	0.4183	< 0.0001	0.9416
Height year 2	< 0.0001	< 0.0001	0.0520
Length of leader 2	< 0.0001	< 0.0001	0.2854
Height year 3	< 0.0001	< 0.0001	0.0411
Length of leader 3	< 0.0001	0.2435	0.1229

**Table 6:** Effects of soil type class on debarked area (cm^2^), height (cm) and length of leading shoot (Leader,cm) three growing seasons after planting

Soil type class	Debarked area	Height	Leader
Undisturbed	9.2a	57.2a	14.8a
Processed	5.1b	64.5b	18.3b
Mixed	3.1c	64.3b	18.8b
Mineral soil	1.9d	67.3b	19.4b

Different letters within columns indicate significant differences

### Effects of site preparation on seedling height

At the time of planting all seedlings had similar heights (Table 5). During the experimental period seedling height and length of leader (average of all living seedlings), were significantly affected by site preparation and insecticide treatment (Table 5, Fig. 5). After the first growing season, insecticide-treated seedlings were larger with longer leaders than the others. After 2 years, all three site preparation treatments also had a positive effect on seedling height and length of leader, and the effect was stronger after 3 years. A significant interaction between the site preparation and insecticide treatments was also detected for seedling height, as site preparation had a stronger positive effect on the insecticide-treated seedlings than on those not treated with an insecticide. No significant differences in seedling height between the three site preparation techniques were detected, but insecticide-treated seedlings planted following preparation using the Karl-Oskar unit tended to have the greatest mean height and length of leader after 3 years (Fig. 5). There were no effects of insecticides on length of leader during the third year, but seedling height was still greater. This is probably due to the fact that the insecticides were no longer active at this point and also due to lower level of damage by pine weevil the third year.

### Effects of soil type class on seedlings’ debarked area and height

Soil type class had a significant effect on the accumulated debarked area 3 years after planting (*p* < 0.0001). It declined with increases in the amount of mineral soil in the planting spot, from undisturbed humus through disturbed humus and a mixture of mineral soil and humus to pure mineral soil (Table 6). Seedling height and length of the leading shoot after 3 years were also affected by soil type class (*p* < 0.0001 for both), but the only significant differences were that seedlings in planting spots with undisturbed soil had lower heights and shorter leading shoots than those in other soil type classes (Table 6).

## Discussion

It is well known that mechanical site preparation resulting in pure mineral soil surrounding seedlings reduces frequencies of attack by pine weevils compared to planting in unprepared soil or a mixture of humus and mineral soil (Lekander and Söderström 1969; Petersson and Örlander 2003; Nordlander et al. 2005; Petersson et al. 2005; Luoranen et al. 2017). Our results show that in the region for the experiment both plant survival and growth were affected by the amount of mineral soil surrounding the seedlings. Various site preparation techniques have different ability to achieve high quality planting spots and it also varies with conditions on the site. After preparation using a Bracke T26, most of the planting spots were defined as tracks or patches, regardless of soil moisture class. Tracks and patches consist in the best cases of pure mineral soil which besides reduction of pine weevil damage also increases soil temperature and reduces competition from undesired vegetation but may also decrease access to nutrients due to the removal of the organic humus layer (Hallsby 1995; Simard et al. 2003).

Most planting spots created by the MidiFlex were classified as mounds/berms. Ideally, in mounds, berms and inverted spots a humus layer is buried under a covering of mineral soil (Sutton 1993). Seedlings can undergo stress just after planting if root growth is not sufficient to couple the seedling to available soil water. This stress can be minimized by preparing favourable planting sites with appropriate preparation. Planted seedlings’ roots will then have access to nutrient-rich layers, thereby promoting seedling establishment and root growth (Nordborg and Nilsson 2003). Sutton and Bedford (2001) showed that mounding that created a layer of mineral soil thick enough to reduce ingrowth of vegetation gave better seedling growth than did more thinly capped mounds. In this study the Karl Oskar resulted in the most diverse kinds of planting spots, with seedlings planted in tracks/patches, mound/berms and inverted spots. At mesic sites, most spots it created were inverted, while at moist sites they were mainly mounds/berms, demonstrating the unit’s adjustability in accordance with site conditions.

Petersson et al. (2005) found that seedlings planted in mineral soil had significantly higher survival rates than seedlings planted in soil mixtures. Accordingly, our results clearly indicate that the relative proportions of soil type classes among planting spots created by a site preparation machine may strongly influence seedling survival rates. The Karl Oskar was the most successful unit in this respect, especially when creating inverted spots, 79% of which were predominantly pure mineral soil. Almost half of the planting spots in tracks and berms created by the Bracke T26 were classified as pure mineral soil, while the Midiflex created a lower proportion (22%) of planting spots with pure mineral soil. Other types of substrates, particularly disturbed humus, probably provide more suitable hiding places, thereby increasing risks of pine weevil feeding on seedlings (Björklund et al. 2003). Thus, our findings clearly highlight the importance of using a site preparation technique that would create the highest proportion of planting spots with pure mineral soil according to specific site conditions.

Of the types of site preparation considered in this study, the distance to humus only exceeded 10 cm (and thus provided close to maximal protection in this respect) in inverted spots created by the Karl Oskar In mounds created by the Bracke T26 and Karl Oskar the distance to humus reached around 7 cm, and in other types of planting spot the distances were shorter. The area debarked by pine weevils reportedly decreases with increasing distance to humus, but only weakly at distances > 10 cm (Örlander and Nordlander 1998; Nordlander et al. 2000).

The height of the planting spots (topography), well described by Sutton (1993) differs between site preparation treatments. As expected, those in tracks and patches were at a similar level to the surrounding ground, while those in mounds and berms were somewhat elevated. Inversion is intended to create planting spots on the same level as the surrounding untreated ground (Örlander et al. 1998; Hallsby and Örlander 2004).

Several studies shows that experience and motivation of the operator influences the efficiency and quality of the work. Karl-Oskar is probably the technique where you have the most possibilities to affect the result. Different operators work differently and might, for example, compress the soil in the planting spot to varying degrees with the aggregate. This might be one reason why the inverted spots created in this study were somewhat elevated. The Bracke T26 created the highest mounds, probably because mounds created by the Midiflex and Karl-Oskar (but not the Bracke T26) are compressed by the unit per se or by the rear bogie.

Structural characteristics of the ground, such as stoniness, will affect the quality of the planting and site preparation (Arnkil and Hämäläinen 1995; Luorananen et al. 2011). On very stony soils, disc trenching with the Bracke T26 created a higher proportion of spots with pure mineral soil than the Karl-Oskar. Johansson (2016) claims that disc trenching or excavator provide the most approved planting spots on stony sites, which is in agreement with Sundblad (2009) who agrees that patching with the excavator based Karl-Oskar is a suitable method for such sites. This warrants consideration when choosing a site preparation method and machine, particularly for very stony sites.

Without site preparation, the seedling mortality rate was very high, almost 80% after three growing seasons, and the main cause was damage by pine weevil. These findings are consistent with observations of seedlings planted on fresh clear-cuts in southern Sweden in several previous studies (Nordenhem 1989; Örlander and Nilsson 1999; Day et al. 2004). When seedlings are not provided with any kind of protection, such as an insecticide, site preparation has substantial effects (Petersson and Örlander 2003; Nordlander et al. 2011). In our study, site preparation by the Bracke T26 and Karl Oskar units resulted in both the lowest mortality rates of untreated seedlings and highest proportions of planting spots with pure mineral soil.

During the experimental period, most seedlings were attacked by pine weevils, but not necessarily killed or severely damaged. During the first growing season, both the site preparation and insecticide treatments significantly reduced frequencies of attacked seedlings and debarked areas. In contrast, during the second year, seedlings treated with the insecticide before planting tended to be attacked more frequently, and debarked more severely, than untreated seedlings. Wallertz and Petersson (2011) recorded the same tendency for seedlings that were protected the first year. However in both the present and in the cited study, the increased damage by pine weevil the second year did not lead to increased mortality of the seedlings. This may be due to a negative effect of early pine weevil attacks on the early seedling establishment. Treatments that postpone the start of pine weevil feeding may thus have a positive effect on the seedlings initial establishment which can explain the enhance ability of these seedling to withstand damage during the second year.

Several studies have also shown that the tolerance of seedlings to pine weevil damage is influenced by their stem base diameter (Örlander and Nilsson 1999; Thorsen et al. 2001). In this study only the seedlings’ height was measured, but the result showed that insecticide-treated seedlings were taller (and thus probably thicker) after the first season than those who had not received any insecticide treatment.

### Practical implications

In general, the results from the present study highlights the importance of choosing the right method of site preparation for a given site. Disc trenching; either with Bracke T 26 or Midiflex, creates relatively homogenous characteristics of planting spots over the prepared area. In contrast, Karl Oskar due to its flexibility can select the best site preparation method for each individual potential planting spot by using either inverting, mounding or patching. The advantage of this is reflected in the results since Karl Oskar overall created the highest number of high quality planting spots. On the other hand; on very stony sites disc trenching has the advantage of producing more acceptable planting spots due to its continuous operational mode. In the results this is demonstrated by the fact that Bracke T26 produced highest number of planting spots on very stony sites.
